# Improving Stability of Tear Film Lipid Layer via Concerted Action of Two Drug Molecules: A Biophysical View

**DOI:** 10.3390/ijms21249490

**Published:** 2020-12-14

**Authors:** Petar Eftimov, Agnieszka Olżyńska, Adéla Melcrová, Georgi As. Georgiev, Philippe Daull, Jean-Sebastien Garrigue, Lukasz Cwiklik

**Affiliations:** 1 Department of Cytology and Embryology, Faculty of Biology, University of Sofia, 1504 Sofia, Bulgaria; peftimov@uni-sofia.bg; 2 J. Heyrovský Institute of Physical Chemistry, Czech Academy of Sciences, Dolejškova 3, 18223 Prague, Czech Republic; agnieszka.olzynska@jh-inst.cas.cz (A.O.); a.melcrova@rug.nl (A.M.); 3 iBB—Institute for Bioengineering and Biosciences, Complexo Interdisciplinar, IST, Universidade de Lisboa, 1649-004 Lisbon, Portugal; 4 SANTEN SAS, Novagali Innovation Center, 1, rue Pierre Fontaine, Bâtiment Genavenir IV, CEDEX, F-91458 Evry, France; philippe.daull@santen.com (P.D.); jean-sebastien.garrigue@santen.com (J.-S.G.); 5 Institute of Organic Chemistry and Biochemistry, Czech Academy of Sciences, 16610 Prague, Czech Republic

**Keywords:** tear film, meibum, tear film lipid layer, dilatation rheology, fluorescence microscopy, molecular dynamics, Langmuir trough

## Abstract

The tear film at the ocular surface is covered by a thin layer of lipids. This oily phase stabilizes the film by decreasing its surface tension and improving its viscoelastic properties. Clinically, destabilization and rupture of the tear film are related to dry eye disease and are accompanied by changes in the quality and quantity of tear film lipids. In dry eye, eye drops containing oil-in-water emulsions are used for the supplementation of lipids and surface-active components to the tear film. We explore in detail the biophysical aspects of interactions of specific surface-active compounds, cetalkonium chloride and poloxamer 188, which are present in oil-in-water emulsions, with tear lipids. The aim is to better understand the macroscopically observed eye drops–tear film interactions by rationalizing them at the molecular level. To this end, we employ a multi-scale approach combining experiments on human meibomian lipid extracts, measurements using synthetic lipid films, and in silico molecular dynamics simulations. By combining these methods, we demonstrate that the studied compounds specifically interact with the tear lipid film enhancing its structure, surfactant properties, and elasticity. The observed effects are cooperative and can be further modulated by material packing at the tear–air interface.

## 1. Introduction

The ocular surface is covered by a thin film of tears structured in three layers: a mucin layer, the aqueous phase of the tear film (TF), and the TF lipid layer (TFLL) [1,2]. Immediately adjacent to the corneal and conjunctival epithelial cells, the mucins layer allows for the aqueous phase of the TF to adhere to the otherwise hydrophobic cell membrane. This aqueous phase provides the necessary environment to protect and nurture the epithelial cells present in the avascular and transparent cornea. At the air/TF interface, the TFLL contributes to the stabilization of the TF by decreasing its surface tension and improving its viscoelastic properties [3]. The destabilization of the TF and its rupture is one of the entry points to the dry eye vicious cycle [4] that leads to dry eye disease (DED). Indeed, tear film instability is one of the major factors listed in the TFOS DEWS II revised definition of DED [5]. A thick and stable TFLL was demonstrated to be necessary for a stable TF [6]. The vast majority of DED patients (>80%) have meibomian gland dysfunction (MGD) [7,8]. Meibomian glands are localized within the eyelids and are responsible for the secretion of the nonpolar and some of the polar lipids that structure and constitute the TFLL [9]. In MGD patients, both the quality and the quantity of the secreted lipids are altered, resulting in a thinner and instable TFLL, hence in an unstable TF characterized by short tear film breakup times (TFBUTs). Oil-in-water emulsions supplying nonpolar lipids [10] and surface-active agents at the air/tear surface were demonstrated to improve the clinical signs and symptoms of DED [11,12] and MGD patients [13] with significant improvements of TFBUTs. A specific role of surfactant species in such oil-in-water emulsions was found to be responsible for their favorable interactions with meibomian gland secretion (MGS) [14]. The aim of the present study is to explore further and gain a full understanding of the interactions of some specific surface-active compounds present in the oil-in-water emulsion with MGS. Namely, we investigate two components targeted to the TFLL/aqueous tear interface: (i) cetalkonium chloride (CKC), a long chain (C-16) cationic lipid thought to match with acyl chains of meibomian lipids, and (ii) poloxamer 188 (P188), which is often used as a pharmaceutical ingredient. Both molecules are surface-active and hence may act in a polar lipid-like fashion and therefore can potentially alter the TFLL properties and TF stability [10]. This is particularly relevant in the context of MGD, where polar lipid deficiency is assumed. We use a multi-scale approach employing various models of TFLL, ranging from Langmuir surface balance experiments on human MGS through to measurements using synthetic lipid films, down to molecular-level simulations employing in silico models of TFLL.

The study of human meibum films (the major source of lipids for the tear film lipid layer) with Langmuir surface balance which allows for blink-like deformations has proven historically as a great way to study tear lipids at a physiologically relevant deformation regime while gaining the advantage of precisely controlled conditions [3]. Although there is a certain level of simplification compared to the ocular surface, the tradeoff has proven relevant, and the surface chemistry approach has agreed with or predicted the surface properties of tear lipids in vivo [15,16,17,18,19] as well as the compatibility between the pharmaceuticals and tear film in vivo [20,21,22,23]. The synthetic lipid system, although simplified, allows for direct comparison with both meibum films experiments and molecular dynamic simulations, which would have been unachievable with the complex meibomian layer consisting of hundreds of lipid and protein species [24]. As the meibomian and the synthetic lipid systems are in good experimental agreement, this allows getting some further molecular information from the simulation. Although the system is simplified, it has the advantages mentioned above. The combination of the three techniques allowed us to reveal that the observed effects are lipid-centered and originate from specific interactions of CKC and P188 with the TFLL. They have a cooperative character and can be modulated by material packing at the tear–air interface.

## 2. Results and Discussion

### 2.1. Meibomian Gland Secretion

**Figure 1:**
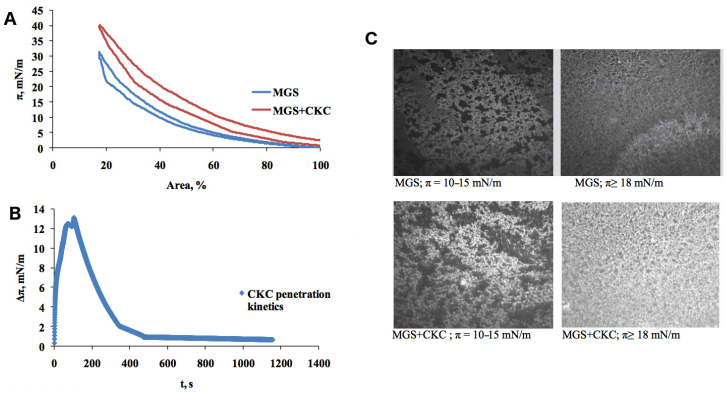
Meibomian gland secretion influenced by cetalkonium chloride (CKC). Lateral pressure–surface area isotherms of meibomian gland secretion (MGS) and MGS in the presence of 3.6 × 10^−7^ M CKC (**A**), a single compression–decompression experiment data are presented; penetration kinetics of 3.6 × 10^−7^ M CKC to MGS film pre-equilibrated at 18 mN/m (**B**); Brewster Angle Microscopy images (500 × 500 μm field of view) of MGS and MGS in the presence of 3.6 × 10^−7^ M CKC (**C**).

To assess the influence of CKC presence on human MGS films, lateral pressure–surface area isotherms were measured using a Langmuir trough. Figure 1A shows isotherms collected for pure MGS and MGS in the presence of 3.6 × 10^−7^ M CKC in the aqueous subphase. In pure MGS, the isotherm represents a typical feature of surfactant films as the pressure rises non-linearly upon compression. No phase transitions are visible, and there is a relatively small hysteresis. It can be concluded that the isotherm shows that MGS behaves as a regular surfactant film at the water–air interface. An addition of CKC to the water subphase results in the overall shift of the isotherm toward larger lateral pressures (and a larger surface area). This transition is similar for most of the area values, and it is somewhat less pronounced in large areas. The shift shows that even at the very low concentration of 3.6 × 10^−7^ M, CKC enhances the MGS layer’s surfactant properties. The elevated lateral pressures along the isotherm suggest that CKC molecules penetrate MGS film, increasing the molecular packing.

To further investigate this incorporation phenomenon, we measured CKC penetration kinetics to MGS film pre-equilibrated at 18 mN/m, corresponding to the formation of duplex film free of monolayer patches similar to the TFLL structure in vivo [25]. The resulting kinetic data are presented in Figure 1B. Although at the investigated concentration (3.6 × 10^−7^ M) CKC has almost no effect on the surface tension of the pure air/water interface (see Figure S1 in the Supplementary Materials), it strongly interacts with MGS and initially, within first 100 s, raises π by 15 mN/m; then, π gradually relaxes to the initial value within ≈1000 s. Such transient perturbations of the surface pressure of MGS films were also observed in the presence of other substances that alter the polar lipid organization at the aqueous/TFLL interface in the period prior to re-equilibration of the film structure to occur [23].

In Figure 1C, bare MGS films and MGS in the presence of CKC are visualized using Brewster angle microscopy (BAM). The imaging, showing increased image brightness in the presence of CKC, demonstrates that CKC has a strong effect on the texture of MGS layers, resulting in increased packing density and film thickness. It is particularly clearly seen that in the presence of CKC, the dark (monolayer) patches interspersed among the thick reflective islands essentially disappear, and thicker (gray) regions with small bright spots within are observed. These results correspond well with both isotherms in terms of the increased packing. On the other hand, the increase of film thickness can be possibly related to the relaxation of the lateral pressure with time observed in the kinetic data (Figure 1B), where the pressure reduction can occur because of a relatively slow buildup of thick film induced by CKC.

**Figure 2:**
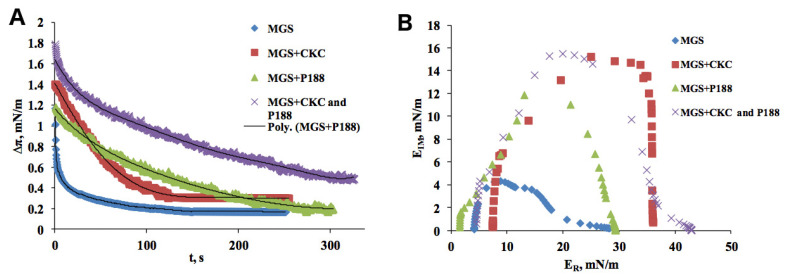
Rheological properties of MGS under the influence of CKC and poloxamer 188 (P188). Relaxation transient (points) and Maxwell rheological equations (lines) of the MGS layer over pure saline solution subphase (physiological saline solution buffer (PBS) pH 7.4), MGS layer over subphase containing 3.6 × 10^−7^ M CKC, MGS over 10^−6^ M P188, and MGS in the presence of 10^−6^ M P188 + 3.6 × 10^−7^ M CKC (**A**); Cole–Cole plot for viscoelastic parameters of the investigated films of MGS, MGS layer over subphase containing 3.6 × 10^−7^ M CKC, MGS over 10^−6^ M P188, and MGS in the presence of 10^−6^ M P188 + 3.6 × 10^−7^ M CKC (**B**).

To assess the influence of CKC on elastic properties of MGS films, dilatation rheology measurements were performed. In Figure 2A, a time relaxation of lateral pressure is presented for MGS and MGS in the presence of CKC. The differences between the films are clearly visible with slower kinetics in the case of MGS in the presence of CKC. Further characterization of the dilatation rheology data was performed by means of Fourier transformation (see Figures S2 and S3 in the Supplementary Materials), and the resulting Cole–Cole plots are presented in Figure 2B. It can be seen that for all systems, E_R_ > E_IM_, i.e., the elastic component of the complex modulus prevailed over the dissipative term. However, the mechanisms of relaxations appear to be different, as reflected by the different shapes of the Cole–Cole plots. In particular, CKC alone resulted in an increased real part of the complex modulus over the entire frequency range.

**Figure 3:**
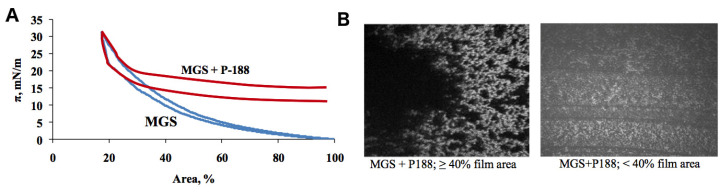
Meibomian gland secretion influenced by P188. Surface pressure/area isotherms of MGS pure and over subphase containing 10^−6^ M P188 (**A**); BAM micrographs (500 × 500 μm field of view) recorded under the lateral pressure prior to squeezing out of P188 (**B**).

In Figure 3, isotherms measured for MGS and MGS over the subphase containing 10^−6^ M P188 are presented. The shift toward higher pressures (≈18 mN/m) at the larger surface areas demonstrates that P188 penetrates the MGS layer. As the system is laterally compressed, the difference between pure MGS and MGS + P188 is diminished. Such behavior indicates that P188 gets squeezed out; this occurs at the pressure of 23 mN/m. BAM micrographs corresponding to the isotherm region prior to being squeezed out are depicted in Figure 3B. They show that the MGS film in the presence of P188 is somewhat more laterally heterogeneous than in the case of CKC. The corresponding dilatational rheology data are depicted in Figure 2 and the Supplementary Figure S4. Although the mixed film remains primarily elastic, the relative contribution of elasticity decreases, and the storage and dissipative parts of the complex modulus almost equalize at 10^−4^–10^−3^ Hz. The absolute value of the dissipative modulus also increases.

**Figure 4:**
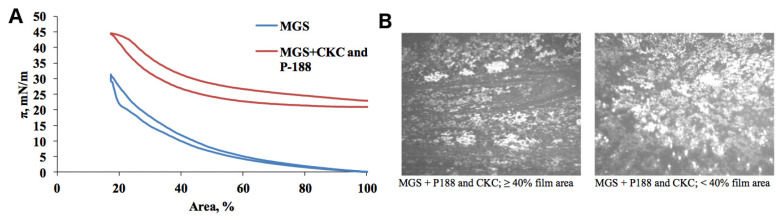
Meibomian gland secretion influenced by P188 and CKC. Surface pressure/area isotherms of MGS pure and over subphase containing 10^−6^ M P188 + 3.6 × 10^−7^ M CKC (**A**); BAM micrographs (500 × 500 μm field of view) of MGS over subphase containing 10^−6^ M P188 + 3.6 × 10^−7^ M CKC (**B**).

The behavior of MGS film spread on a mixed subphase containing 10^−6^ M P188 + 3.6 × 10^−7^ M CKC can be analyzed using the isotherms shown in Figure 4A. At all surface area values, the MGS + CKC + P188 isotherms are shifted toward higher pressures. This process is similar to the case of MGS + CKC and indicates incorporation of the subphase material in the MGS film. However, the extent of the pressure shift is more similar to that of MGS + P188. This behavior indicates that both types of the subphase molecules, CKC and P188, incorporate in the MGS film. Notably, as the pressure increases, the pressure shift, although somewhat diminished, is still present. This is contrary to the MGS + P188 system behavior depicted in Figure 3 and proves that in the presence of CKC, the incorporation of P188 is, to some extent, maintained upon film compression.

The BAM micrographs presented in Figure 4B reveal that in the presence of CKC, the incorporation of P188 is maintained upon film compression. The dilatational rheology data shown in Figure 2 demonstrate that the mixture of 10^−6^ M P188 + 3.6 × 10^−7^ M CKC strongly enhances the surfactant properties of MGS layers. The effect on maximum surface pressures is stronger than the individual impact of CKC or P188.

### 2.2. Synthetic Lipid Films

Synthetic lipid films were used to understand further the interactions of CKC and P188 with lipids at the water–lipid interface. Two synthetic systems were employed. First, measurements using purely polar 1-palmitoyl-2-oleyl-sn-glycero-3-phosphocholine (POPC) lipid monolayers were performed to understand the interactions at the very water–lipid interface mimicking the boundary between the aqueous TF subphase and the polar part of the TFLL. Second, mixed films composed of polar POPC and nonpolar TO were studied to understand the interaction of CKC and P188 with the multilayer lipid films that model the relatively thick TFLL. In both cases, the lipid film was deposited over PBS subphase, whereas CKC and/or P188 were added to the aqueous subphase, and the lateral pressure–surface area isotherms were measured. Furthermore, during film compression, epifluorescence images were continually recorded.

**Figure 5:**
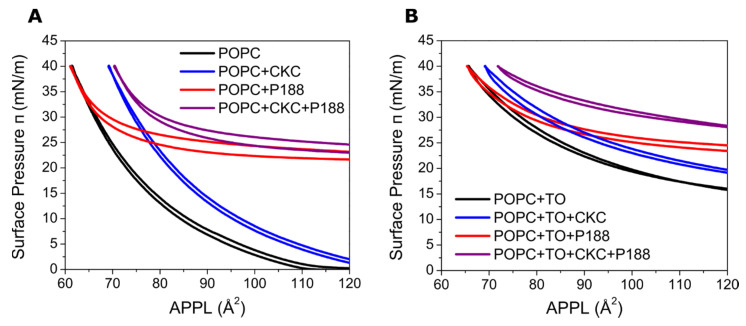
Surface pressure–area per polar lipid (APPL) isotherms of 1-palmitoyl-2-oleyl-sn-glycero-3-phosphocholine (POPC) (**A**) and POPC + triolein (TO) (**B**) films measured over pure subphase (black) and subphase containing P188 (red), CKC (blue), and the mix of P188 and CKC (violet).

Figure 5A shows the isotherms of the POPC monolayer deposited over the pure PBS subphase and the subphase containing CKC (3.6 × 10^−7^ M) and/or P188 (10^−6^ M). All isotherms show the typical behavior of surface films with lateral pressure increasing with a reduced surface area. No phase transition was observed and the hysteresis was insignificant in each case. The presence of CKC in the subphase leads to the shift of the isotherm toward higher pressures, and this effect is present at all studied compressions. This behavior resembles that observed for MGS + CKC film (Figure 1A) and indicates that CKC incorporates into the POPC monolayer. In the case of P188 present in the subphase, the isotherm is also shifted toward higher pressures, but the shift disappears when the film is laterally compressed. This phenomenon is analogical to that observed in the MGS + P188 system (Figure 3A) and shows that P188 incorporates into laterally relaxed POPC film at ≈24 mN/m but is squeezed out when the film is compressed to ≈32 mN/m. These values of the pressure are by 6–9 mN/m larger than those observed in the MGS + P188 case. This may be rationalized by the fact that the synthetic systems consist only of lipids that interact directly with both surfactants, whereas the natural MGS also contains non-lipid components. When a mixture of CKC and P188 is present in the subphase, the effects of interactions sum up. The mixture penetrates the film at the pressure ≈ 24 mN/m and stably increases its surfactant properties. A synergy is observed between CKC and P188 as their influence is more pronounced when they both are present in the aqueous subphase. Overall, this behavior is analogical to that observed in the case of MGS + CKC + P188 films (Figure 4A).

The isotherms measured for the mixed POPC + TO films in the presence of CKC and/or P188 are presented in Figure 5B. The two-component POPC + TO film at the pure water subphase was characterized in our previous study and was proposed as a minimalistic synthetic model of the tear film lipid layer [26]. In the measured POPC + TO film, the isotherm shows no phase transition and is shifted toward higher pressures than in the case of the mono-component POPC film. These conditions correspond to the film with some of the triolein (TO) molecules incorporated into the POPC monolayer, while the rest of the TO resides atop of this film [26]. When CKC is added to the aqueous subphase, the isotherm is in overall shifted toward higher pressures, hence suggesting that CKC incorporates in the film. In the case of P188, the shift disappears at ≈32 mN/m, demonstrating that P188 can be initially incorporated into the film but is squeezed out under high lateral compressions. Eventually, in the case of CKC and P188 present together, a synergic combination of both effects is observed.

Epifluorescence images were collected for both POPC and mixed POPC + TO films spread at a neat buffer as well as a buffer containing CKC and/or P188 (see Figures S5–S9 in the Supplementary Materials). The film was labeled with two fluorescent probes, one preferentially located in the polar lipid environment and the other probing the nonpolar region of the film. The analysis of the images shows that while the incorporation of CKC in the POPC film does not cause noticeable changes in its structure (Figure S5), P188 leads to the formation of lateral inhomogeneities, in particular at lower pressures (Figure S6). In the case of the mixed POPC + TO films, the influence of CKC but also P188 is not pronounced in the imaging. Namely, the system behaves as a pure polar–nonpolar mixed film, in analogy to the hybrid systems investigated in our previous study [26]. This is despite the fact that the isotherms analysis shows that the adsorption of both species alters the system. It can be rationalized by the fact that in the mixed POPC + TO film, incorporation of the surfactants is overall smaller when comparing with a pure POPC monolayer, as evidenced by a relatively small increase of lateral pressures at the corresponding isotherms (compare Figure 5A,B).

### 2.3. MD Simulations

Molecular dynamics simulations were employed for gaining a molecular-level view of the lipid films in the presence of CKC and P188. The in silico model of TFLL that we introduced in our previous studies was used here [27,28,29]. The model consists of a multicomponent film of both polar and nonpolar lipids spread at the water–air interface, as described in the Materials and Methods section. MD simulations were performed at two different values of area per polar lipid (APPL) to address the system’s behavior under different lateral film compression with pure lipid films and films supplemented with CKC and/or P188. The aim was to provide a molecular-level rationalization of observations obtained in Langmuir trough experiments. APPL = ≈90 Å^2^ was chosen to model a laterally compressed film, and the value of ≈350 Å^2^ was used to represent a laterally relaxed film. As can be seen at the experimental isotherms (Figure 5B), in the synthetic polar–nonpolar films, APPL of 90 Å^2^ corresponds to a relatively packed film in which still both CKC and P188 interact with lipids; while APPL of 350 Å^2^ (not shown in Figure 5B) represents a relaxed film.

**Figure 6:**
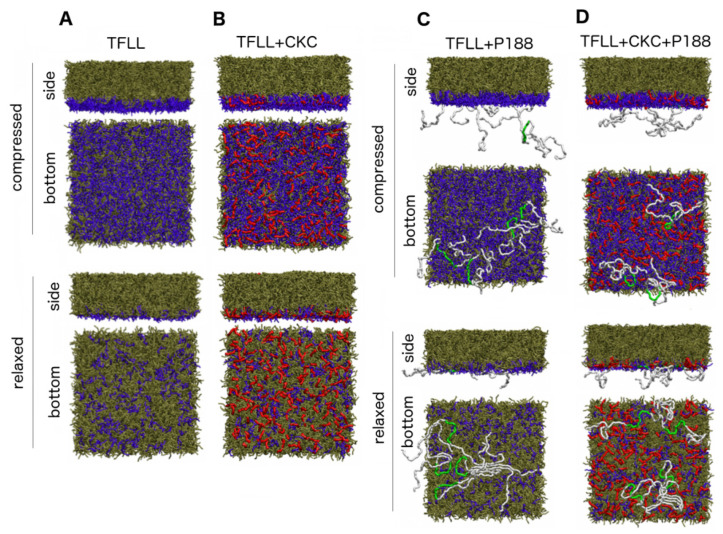
Simulation snapshots obtained in the course of simulation of the tear film lipid layer (TFLL) model (**A**), and the model in the presence of CKC (**B**), P188 (**C**), and the mixture of both CKC and P188 (**D**). In each case, snapshots for the simulation performed at area per polar lipid APPL = 91 Å^2^ (designated as compressed) and 354 Å^2^ (designated as relaxed) are presented. Side (upper panel) and bottom (lower panel) views of the lipid films are depicted for each system, the bottom views are taken from the direction of the water phase. Nonpolar lipids are shown in tan, polar lipids are shown in blue, CKC are shown in red, P188 hydrophobic cores are shown in green, and P188 hydrophilic tails are shown in white. Water molecules are omitted for clarity. Visualization was performed using VMD 1.9 software [32].

In MD simulations, there was a clear separation between water and the lipid film, and between the polar lipids monolayer and a multilayer of nonpolar lipids. This behavior is visible in simulation snapshots shown in Figure 6 and is further quantified in the density profiles shown in Supplementary Materials (Figures S10–S12). Notably, such a separation between the polar and nonpolar lipid layers, together with a continuous character of the polar layer, is essential for the molecular-level stability of TFLL, as demonstrated in earlier studies [27,30,31]. Despite the overall lipid–water separation, in the laterally relaxed system, the polar layer is not continuous with small transient pores formed, leading to increased contact between the nonpolar lipids and water. This is evidenced in the snapshots as well as by the appearance of a peak at the triglyceride density profiles that emerges in the region overlapping with water.

MD simulations that included CKC were started with the CKC molecules prearranged in the polar monolayer. This protocol was motivated by the previous experimental observations that CKC incorporates into polar films, but that kinetics of this process is slow; hence, direct simulations of CKC incorporation would be unfeasible. Simulations demonstrated that CKC is stably located in the polar lipid layer (see Figure 6B and density profiles in Figure S11 in the Supplementary Materials) with its polar headgroups oriented toward the water. We observed that on the timescale of microseconds probed in the present simulations, only an insignificant fraction (few CKC molecules) desorb toward the nonpolar sublayer. Due to a shorter length of CKC (sixteen carbon atoms) than that of polar lipids (such as POPC with eighteen carbon atoms in the longer acyl chain), CKC is somewhat buried in the polar headgroup region. Hydrophobic tails of CKC are aligned in parallel with the tails of polar lipids. Importantly, it can be observed that the presence of CKC in the polar layer reduces the number of water–nonpolar lipids contacts in the systems with low lateral compression. This behavior is evidenced by a less pronounced triglyceride density in the region overlapping with water in the case of CKC-containing film (the triglyceride peak located at ≈0.5 nm from the interface in the relaxed films is smaller in Figure S11 than in Figure S12 in the Supplementary Materials). A further inspection of MD trajectories revealed that this is due to the fact that CKC spreads at the water–lipid interface relatively homogeneously, compensating for the low lateral packing of polar lipids and hence suppressing water–nonpolar lipids contacts. The homogenous spreading of CKC can be rationalized by its positive charge that leads to repulsion between individual molecules.

The behavior of P188 in the vicinity of the TFLL model is determined by the presence of both hydrophobic and hydrophilic patches in P188 molecules. As shown in the snapshots in Figure 6C as well as in the Supplementary Figure S12, at APPL = 91 Å^2^, hydrophobic cores of P188 molecules predominantly adsorb at the water–lipid interface. In fact, they are buried below the polar headgroups of polar lipids (e.g., cholines). At the same time, hydrophilic tails remain partially adsorbed but mostly reside in the water phase. Significantly, as the APPL increases, the adsorption of polar tails at the water–lipid boundary is enhanced. Similar to CKC, the presence of P188 reduces the peak of triglycerides next to the water phase, but this effect is weaker for P188 than for CKC. Further analysis of MD trajectories demonstrated that P188 adsorbs mostly in pores formed in the deficient polar layer. However, in contrast to CKC, P188 does not incorporate in-between other polar lipids because of its big molecular size. Rather, molecules of P188 adsorb flat at the interface (see the snapshots in Figure 6C). The adsorption of predominantly hydrophobic molecular cores of P188 (shown in green) at low pressure is enhanced and includes hydrophilic chains (shown in white) as APPL increases. In addition, a flat orientation of the strongly adsorbed P188 at the water–lipid interface is clearly visible. This behavior explains both the reduction of water–nonpolar contacts experienced by the TFLL model upon P188 supplementation and the fact that this effect is weaker than that induced by CKC, which behaves as a regular short-chain surfactant that incorporates between native lipids.

**Figure 7:**
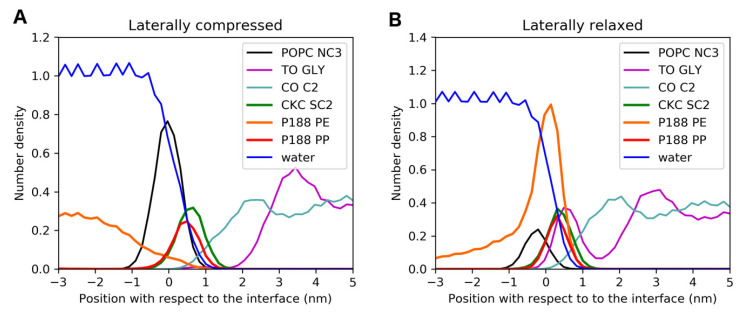
Density profiles of selected components of the TFLL model calculated in simulated films with area per polar lipid (APPL) = 91 Å^2^ (designated as compressed) (**A**), and 354 Å^2^ (designated as relaxed) (**B**) in the presence of both CKC and P188. Number densities of water, POPC polar headgroup (“POPC NC3”), triglyceride glycerol backbone (“TO GLY”), cholesterol oleate short chain terminal (“CO C2”), CKC polar headgroup (“CKC SC2”), P188 nonpolar core (“P188 PE”), and P188 polar tails (“P188 PP”) are depicted. The left-hand side of each graph represents the aqueous tear subphase, whereas the right-hand side corresponds to the nonpolar sublayer of TFLL. The data for the second lipid–air interface present in the simulation box (not shown here) are virtually the same. For comparison, the density profiles of the not supplemented system and the systems supplemented with CKC and P188 only are shown in Supplementary Figures S10–S12.

The simulation of the TFLL model in the presence of both CKC and P188 reveals that CKC behaves similarly as in the absence of P188, i.e., it adsorbs at the interface, incorporates in-between native polar lipids, and fills the pores formed in the polar sublayer. This is evidenced in the snapshots in Figure 6D and in the density profiles (green curves) in Figure 7. In contrast to CKC, P188 in the presence of CKC behaves differently than in the P188-only case. More specifically, while nonpolar moieties of P188 are still adsorbed at the water–lipid interface (red curves in Figure 7), the presence of CKC to a significant extent diminishes the adsorption of polar P188 tails at low APPL (orange curve in Figure 7). However, as APPL increases, the polar tails adsorb at the interface. Hence, a mode of P188 adsorption is modulated by lateral compression. This can be rationalized by steric reasons as CKC effectively fills up the gaps formed in the discontinuous polar lipid layer that otherwise would be occupied by P188 tails. Nevertheless, the observed well-pronounced adsorption of polar patches of P188 molecules at the interface in the presence of CKC is responsible for the synergic action of CKC and P188 in the reduction of water–nonpolar lipid contacts.

It should be noted that the observed behavior of P188 regarding its relatively strong adsorption to the water–lipid interface via its polar patches and a modulated adsorption via nonpolar patches is interesting in light of tear proteins. Namely, some of the surface-active proteins present in the aqueous phase of TF, such as the lysozyme, can adsorb to the lipid layer via their polar parts [29]. We can hypothesize that nonpolar patches of such proteins may act similarly to the nonpolar parts of P188 and undergo a modulated adsorption depending on the lateral packing of the lipid film. Hence, it is plausible that in the presence of CKC, some of the tear proteins may act cooperatively with CKC and stabilize the lipid film. Such behavior would be beneficial as the protein concentration in TF is constant, while the water-soluble P188 undergoes a relatively fast removal from TF upon instillation.

## 3. Materials and Methods

### 3.1. Meibomian Gland Extracts

#### 3.1.1. Compression Isotherms

Meibomian gland extracts were provided by Prof. Norihiko Yokoi, Kyoto Prefectural University of Medicine, with Permission by the Ethics Committee and in agreement with the tenets of the Declaration of Helsinki. The Langmuir surface balance µTrough XS, area 135 cm^2^, volume 100 mL (Kibron, Helsinki, Finland) was used to collect the surface pressure–area (π-A) isotherms utilizing the Wilhelmy wire probe method (instrumental accuracy 0.01 mN/m) [21,22,23]. Physiological saline solution buffer (PBS, pH 7.4), pure and with the inclusion of 10^−6^ M P188 and/or 3.6 × 10^−7^ M CKC, respectively, was loaded as a trough subphase. Microsyringe (Hamilton Co., Reno, NV, USA) was used to deposit human MGS (35 µL of 1 mg MGS/mL CHCl_3_) at the air/saline interface. The trough was positioned under an acrylic cover protecting the surface from dust and suppressing the evaporation of the aqueous subphase. After 15 min provided for chloroform evaporation, the film compression was processed by two symmetrically moving barriers. Dynamic compression–expansion isocycling of the layer was performed at 70 mm/min (with the maximum barrier’s rate without film leakage). Ten consecutive cycles were performed with each sample. After the third cycle, the π(A) curves attained a stationary shape, and those measurements were presented and analyzed. All isotherms were repeated at least four times; the difference between the repetitions was typically less than 2%. The presented isotherms are averaged on at least three measurements. As some minimal noise is normal in complex and heterogeneous films such as MGS, the resulting isotherms are less smooth than those measured for the simpler synthetic lipid films. The experiments were done at 35 °C. The films’ morphology was monitored by Brewster Angle Microscopy (MicroBAM, KSV-NIMA).

#### 3.1.2. Stress–Relaxation Studies via the Small Deformations Method

To gather information about the dilatational viscoelasticity of meibum films, pure and with CKC and P188, the relaxation of the surface pressure was monitored after a small rapid compressional deformation was applied to the surface film. Firstly, the film was compressed to *π*_0_ = 18–20 mN/m. Then, the layer was instantaneously and slightly contracted with a compression step, ∆*A*/*A*_0_ = 5 ± 1% (*A*_0_ is the initial film area, and ∆*A*—area change) [14,33]. The relaxations are presented in (*π_t_* − *π*_0_)/(*π_max_* − *π*_0_) = *f*(*t*) coordinates, where *π_t_* is the momentum value of the surface pressure and *π_max_* is its maximum (starting) value. The dependence of the real, *E_R_*, and imaginary part, *E_IM_*, of the complex dilatational elasticity modulus E*(ν) on frequency, *ν*, was given via Fourier transformation, *F*, of the transients [25]:

$$E^{*}\left(\nu \right)\;=\;E_{R}\left(\nu \right)+iE_{IM}\left(\upsilon \right)\;=\;\frac{F\left\{d∆\pi \left(t\right)/dt\right\}}{F\left\{dlnA/dt\right\}}\;=\;\frac{i6.28\nu }{∆A/A_{0}}\int_{0}^{\infty }∆\pi \left(t\right)\exp\left(-i6.28\nu t\right)dt.$$

Here, *E_R_* represents the elasticity of the film, while *E_IM_* set by the product of *ν* η_d_ (η_d_ is the dilatational viscosity) accounts for the dissipative, viscous properties of the film. The number 6.28 is a brief denotement of the doubled Archimedes constant (2 × 3.14159…). The Fourier analysis of the relaxation transients was performed as previously described [14,33], utilizing commercial Fourier transform software provided by Kibron Inc. (Helsinki, Finland).

If *E_R_* > *E_IM_*, the film is predominantly elastic. On the contrary, if *E_R_* < *E_IM_*, the film is predominantly viscous. The data were subjected to further analysis by constructing their corresponding Cole–Cole plots (i.e., graphs of *E_IM_* vs. *E_R_*). The number of peaks in the Cole–Cole plot shows the number of processes contributing to the relaxation [14,33]. Here, two peaks emerged in the plots, which can be further probed by fitting the raw relaxation transients with the corresponding number of decaying exponents and a plateau, thus yielding:

(1) $$\Delta \pi \;=\;A_{i}\exp\sum \left(-\frac{t}{\tau_{i}}\right)+\Delta \pi_{EQ}$$

where the number of exponent terms (*i*) is equal to the number of peaks in the Cole–Cole plot; A is the pre-exponential factor reflecting the contribution of the individual term to the relaxation; *τ* is the characteristic relaxation time; and Δ*π_EQ_* is the plateau value.

In the framework of the generalized Maxwell model [34], the equation denotes parallel elements in which each exponential term corresponds to a Maxwell spring-and-dashpot element and Δ*π_EQ_* corresponds to a spring element representing the equilibrium elasticity (see Figure S13 in the Supplementary Materials) [14,33]. The relaxation time of a Maxwell element is given by the ratio of its viscosity and elastic modulus (*τ_i_* = *η_i_*/*E_i_*).

### 3.2. Compression Isotherms and Epifluorescence Imaging of Synthetic Lipid Films

The Langmuir film measurements were performed employing a MicroTroughXS setup (Kibron, Helsinki, Finland). A metal alloy trough with the maximum working surface area of 12,331 mm^2^ was used for non-microscopic experiments. In the fluorescence studies, a metal alloy trough with a quartz-glass window mounted on the inverted fluorescence microscope (Olympus, Tokyo, Japan) was applied. The trough was filled with a subphase that was 10 mM phosphate-buffered saline (PBS) (0.137 M NaCl, 0.0027 M KCl, pH 7.4; Sigma-Aldrich, St. Louis, MO, USA) prepared using Milli-Q water (Millipore, Burlington, MA, USA). 1-palmitoyl-2-oleyl-sn-glycero-3-phosphocholine (POPC) (Avanti Polar Lipids, Alabaster, AL, USA) and triolein (TO) (Sigma-Aldrich, St. Louis, MO, USA) were chosen as a representation of polar and nonpolar TFLL lipids, respectively. For epifluorescence imaging, 1,2-dioleoyl-sn-glycero-3-phosphoethanolamine-Atto 633 (DOPE–Atto633) (ATTO-TEC, Siegen, Germany) and 4,4-Difluoro-1,3,5,7,8-Pentamethyl-4-Bora-3a,4a-Diaza-s-Indacene (Bodipy 493/503) (Thermo Fisher Scientific, Waltham, MA, USA) fluorescent probes were mixed in chloroform (spectroscopic grade; Sigma-Aldrich, St. Louis, MO, USA) with POPC and TO, respectively. The lipid to probe molar ratio was 1000:1. The appropriate volume of 1 mM chloroform solution of lipids (POPC with or without TO) was spread over the subphase using a Hamilton microsyringe. Surface pressure–molecular area (π-A) isotherms were recorded employing an ultra-sensitive surface pressure sensor (KBN 315; Kibron, Helsinki, Finland) with the DyneProbe during the symmetrical movement of two barriers controlled by software (FilmWare) provided by the equipment manufacturer. The speed of compression was 10 mm/min (3.926 Å^2^/chain/min). The experiments were performed at 34.5 °C with an accuracy of ±0.5 °C using a temperature control plate placed under the trough, which was connected to a water-circulating thermostat. An acrylic cover box was placed over the trough to slow down subphase evaporation as well as protect from dust and other surface disruptions. Before each measurement, the trough was left uncovered for 7 min to allow for chloroform evaporation. Then, P188 and/or CKC (both provided by Santen, Evry, France) dissolved in PBS were added to the subphase to obtain the final concentrations of 10^−6^ M and 3.6 × 10^−7^ M, respectively. The system was left for 25 min for equilibration. During the measurements employing the microscope, evaporation of the subphase in the trough was counterbalanced by slow continuous refill with the buffer from outside of the barriers using a peristaltic pump (Harvard Apparatus, Holliston, MA, USA). To study the morphology of the lipid films, epifluorescence images were collected applying an air objective PLAN N 40× (NA 0.65, WD 0.6 mm; Olympus, Tokyo, Japan) or water immersion objective UPlanSApo 60× (NA 1.2, WD 0.28 mm; Olympus, Tokyo, Japan). As an excitation source, a mercury lamp was employed. Signal detection was realized using a CCD camera (Olympus, Tokyo, Japan). Two filter cubes, Cy5-A-Basic-000 (single-band 630/38 excitation and 694/44 emission filters; Semrock, Rochester, NY, USA) and U-MWB2 (single-band 475/30 excitation and 525/39 emission filters; Olympus, Tokyo, Japan) were mounted in the light pathway and used interchangeably during measurement. The epifluorescence images were recorded with the acquisition time of 75 ms.

### 3.3. MD Simulations

The simulated systems were constructed in accordance to our previous TFLL simulation studies [27,28,29]. Namely, a film consisting of the mixture of polar and nonpolar lipids was simulated at the water–air interface with and without the addition of CKC and P188 molecules. The polar lipids included 1-palmitoyl-2-oleoyl-phosphatidylcholine (POPC), 1-palmitoyl-2-oleoyl-phosphatidylethanolamine (POPE), *N*-palmitoyl-d-erythro-sphingosine (Cer), and *N*-palmitoyl-d-erythro-sphingosyl-phosphorylcholine (SM); with the molar ratio (POPC—68%, POPE—22%, SM—5%, Cer—5%) mimicking the experimental lipidomic data from a previous study [35]. These polar lipids formed a monolayer at the interface with water. The polar monolayer was covered by a relatively thick nonpolar lipids phase consisting of glycerine trioleate (TO) and cholesteryl oleate (CO) in ≈1:1 molar ratio. Such a lipid arrangement, i.e., a polar monolayer covered by a thick nonpolar layer, is in accord with the currently accepted model of molecular-level organization of TFLL [2]. To simulate the film under different lateral compressions, two simulation variants, differing with the total number of polar lipid in the system, were performed. The numbers of lipids were chosen such that a desired APPL was obtained while the size of the interface was kept constant. Namely, APPL = ≈90 Å^2^ was chosen to model a laterally compressed film, and APPL= ≈350 Å^2^ was used to represent a laterally relaxed film. Exact system compositions are given in the Supplementary Materials (Table S1).

CKC was added to the polar layer in 5.6 × 10^−2^ molar concentration (calculated with respect to the water phase), leading to a CKC to polar lipid molar ratio of 0.27 and 1.05 in the laterally compressed and expanded system, respectively. The adding of CKC directly to the polar lipid layer was performed in order to reduce the simulation time that would be required for the explicit penetration of CKC from water to the lipid phase. CKC stayed in the polar sublayer on the course of simulations with only a few individual CKC molecules being able to penetrate into the nonpolar lipid layer. To neutralize the positive charge of cetalkonium cations, the same number of chloride anions was added to the water phase. P188 was added to the water phase in 1.5 × 10^−3^ molar concentration.

Eight systems (pure lipid films, films in the presence of either CKC or P188, and films in the presence of both CKC and P188; each under high and low lateral pressure) were simulated. Each trajectory was calculated for 2 µs, with the last 1 µs used for analysis (the number of contacts between the individual system’s components was employed as the equilibration criterium). MD simulations were performed using the coarse grain approach. The standard MARTINI coarse grain model was used for the representation of all molecules with an in-house parameterization for missing ones (ceramides and CKC, see the Supplementary Materials). In the case of P188, a MARTINI parameterization derived by Hezaveh et al. was used [36]. MD simulations were performed with GROMACS software suite [37]. Further simulation details are described in the Supplementary Materials.

## 4. Conclusions

We aimed at explaining at the biophysical level an improved stability of human Tear Film observed in clinical conditions [11,38] by exploring the interactions and effects of two drug molecules (CKC and P188) with the TFLL and its components. To this end, we performed a multi-scale study using various models of TFLL studied by various approaches, ranging from experiments on human MGS through to measurements using synthetic lipid films, down to molecular-level simulations employing in silico models of tear lipids layer. By this, we can understand the behavior of the film observed in the experiments as well as rationalize some of the clinical observations. Using MGS, we demonstrated that CKC is a polar lipid-like molecule that stably incorporates in the MGS layer (i.e., TFLL) and enhances its structure, surfactant property, and elasticity. P188, being a water-soluble surfactant, has limited interaction with meibum and gets squeezed out of MGS films at sub physiological surface pressures. However, the mixture of P188 (10^−6^ M) and CKC (3.6 × 10^−7^ M) strongly enhances the surfactant properties of MGS layers. The effect on maximum surface pressures is stronger than the individual impact of CKC or P188.

The observations on MGS films were directly repeated using films composed of synthetic lipids. This demonstrates that the observed effects originate predominantly from interactions of the considered molecules with lipids. The observed phenomena have a cooperative character and can be modified by changing the material packing in the lipid films. Furthermore, we gained a molecular-level rationalization of the observed phenomena. In silico modeling proves that CKC stably incorporates in the polar sublayer of the lipid films while P188 adsorption from the water subphase is modulated by the presence of CKC as well as by lipid film packing.

Hence, it can be assumed that at the ocular surface, the long-term effects of the cationic nanoemulsions are predominantly determined by CKC and the other oil phase compounds of the nanoemulsions, while the benefits of P188 are transient as the molecule is washed out from the TFLL/water interface within a few blinks.

Such a comprehensive multi-level understanding of the phenomena related to drug–tear lipid layer interactions is important both from the basic scientific viewpoint as well as from the perspective of drug design. Namely, by knowing which molecular characteristics of the considered drugs are vital for their interactions with tear lipids and how these interactions translate to film behavior, one can propose or redesign drug molecules to improve their clinically important properties as the stabilizing influence on tear film or resident time at the ocular surface.

## Supplementary Materials

The following are available online at https://www.mdpi.com/1422-0067/21/24/9490/s1, Figure S1: Dependence of CKC adsorption at pure air/water interface on the bulk concentration of CKC; Figure S2: Output of the Fourier transformation of the relaxation transient of pure MGS; Figure S3: Output of the Fourier transformation of the relaxation transient of the MGS layer over the subphase containing CKC; Figure S4: Output of the Fourier transformation of the relaxation transient of the MGS layer in presence of P188; Figure S5: POPC monolayer labeled with DOPE-Atto633 over the subphase containing CKC. Representative epifluorescence images recorded during the compression of monolayer at different surface pressure/APPL; Figure S6: POPC monolayer labeled with DOPE-Atto633 influenced by P188 present in the subphase. Representative epifluorescence images recorded during one compression experiment in different film regions at the surface pressure ≈ 25 mN/m; Figure S7: Epifluorescence images of POPC + TO film over the PBS subphase containing CKC; Figure S8: Epifluorescence images of POPC + TO film over PBS subphase containing P188; Figure S9: Epifluorescence images of POPC + TO film over PBS subphase containing CKC + P188; Figure S10: Density profiles of the selected TFLL model components calculated in simulated films with different area per polar lipid; Figure S11: Density profiles of selected components of the TFLL model calculated in simulated films with different area per polar lipid in the presence of CKC; Figure S12: Density profiles of selected components of the TFLL model calculated in simulated films with different area per polar lipid in the presence of P188; Figure S13: Schematic representation of the employed Maxwell models; MD simulation details; Table S1: Compositions of simulated systems; Force field parameters of the ceramide and CKC molecules.
