# Supplementary material for: Improving Stability of Tear Film Lipid Layer via Concerted Action of Two Drug Molecules: A Biophysical View
Source: Int J Mol Sci. 2020 Dec 14;21(24):9490. doi: 10.3390/ijms21249490 (PMC7764870; doi:10.3390/ijms21249490)
Supplement: Supplementary file 1 [file ijms-21-09490-s001.pdf]

Supplementary Materials

Improving Stability of Tear Film Lipid Layer via Concerted Action of Two Drug Molecules: a Biophysical View

Petar Eftimov,[1] Agnieszka Olżyńska,[2] Adéla Melcrová,[2] Georgi As. Georgiev,[3*] Philippe Daull,[4] Jean-Sébastien Garrigue,[4] Lukasz Cwiklik[2,5,*]

[1] Department of Cytology and Embryology, Faculty of Biology, University of Sofia, Sofia, Bulgaria

[2] J. Heyrovský Institute of Physical Chemistry, Czech Academy of Sciences, Dolejškova 3, 18223 Prague, Czech Republic

[3] iBB - Institute for Bioengineering and Biosciences, Complexo Interdisciplinar, IST, Universidade de Lisboa, Lisbon, Portugal

[4] SANTEN SAS, Novagali Innovation Center, 1, rue Pierre Fontaine, Bâtiment Genavenir IV, F-91458 Evry Cedex, France

[5] Institute of Organic Chemistry and Biochemistry, Czech Academy of Sciences, 16610 Prague, Czech Republic

[*] corresponding authors:

lukasz.cwiklik@jh-inst.cas.cz and georgi.georgiev@tecnico.ulisboa.pt

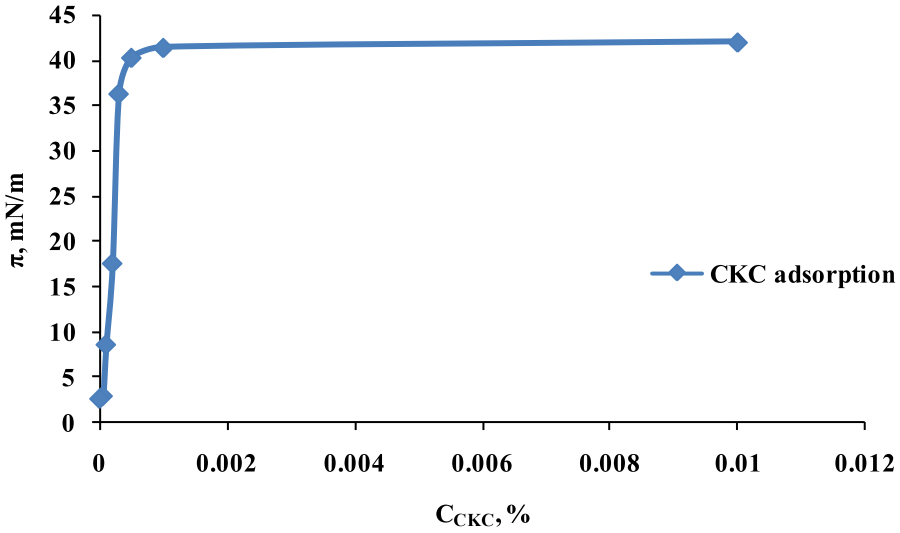

Figure S1. Dependence of CKC adsorption at pure air/water interface on the bulk concentration of CKC. The analysis of the curve with the Gibbs adsorption isotherm yields the area of 41.14 (±12) Å^2 per CKC molecule.

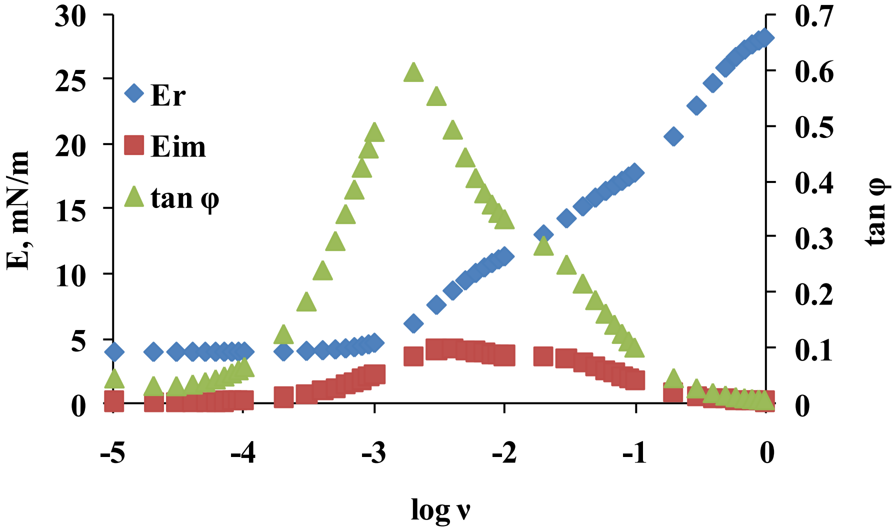

Figure S2. Output of the Fourier transformation of the relaxation transient (Figure 2) of pure MGS.

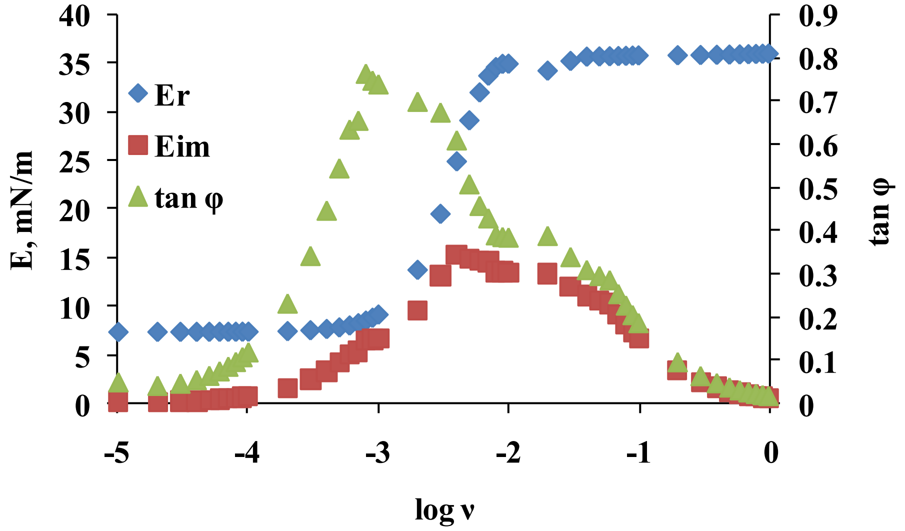

Figure S3. Output of the Fourier transformation of the relaxation transient (Figure 2) of MGS layer over subphase containing 3.6×10^{-7} M CKC.

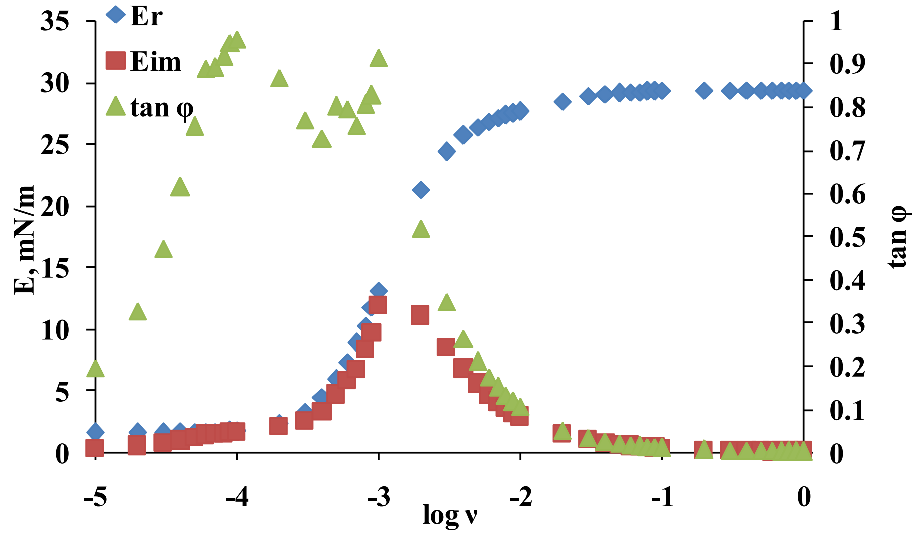

Figure S4. Output of the Fourier transformation of the relaxation transient (Figure 2) of MGS layer in presence of P188.

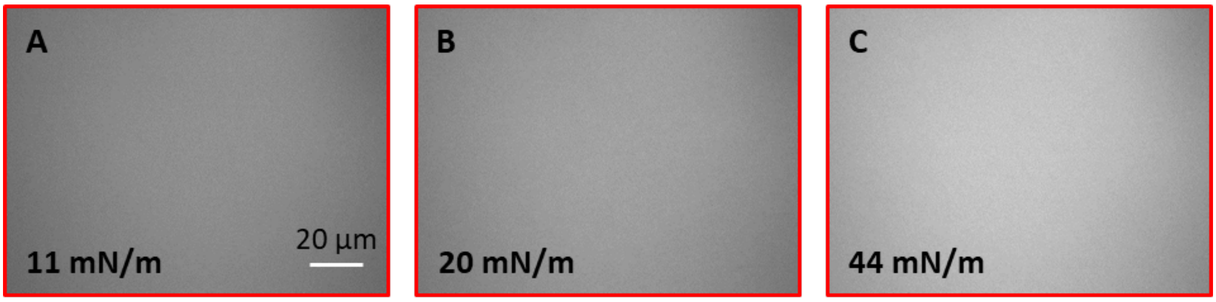

Figure S5. POPC monolayer labeled with DOPE-Atto633 over the subphase containing 3.6×10^{-7} M CKC. Representative epifluorescence images recorded during the compression of monolayer at surface pressure/APPL of 11 mN/m (A), 20 mN/m (B), and 44 mN/m (C).

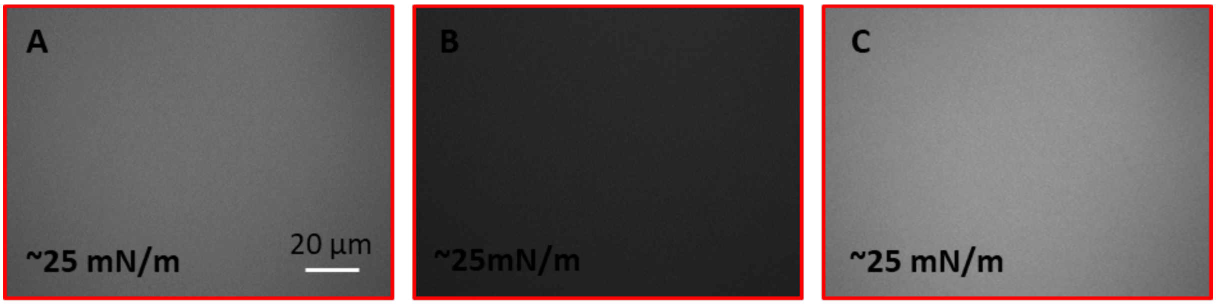

Figure S6. POPC monolayer labeled with DOPE-Atto633 influenced by P188 present in the subphase at the concentration 10^{-6} M. Representative epifluorescence images recorded during one compression experiment in different film regions at the surface pressure ~25 mN/m. The differences in the intensities between the images shows that the film was inhomogeneous in the scale above ~100 μm.

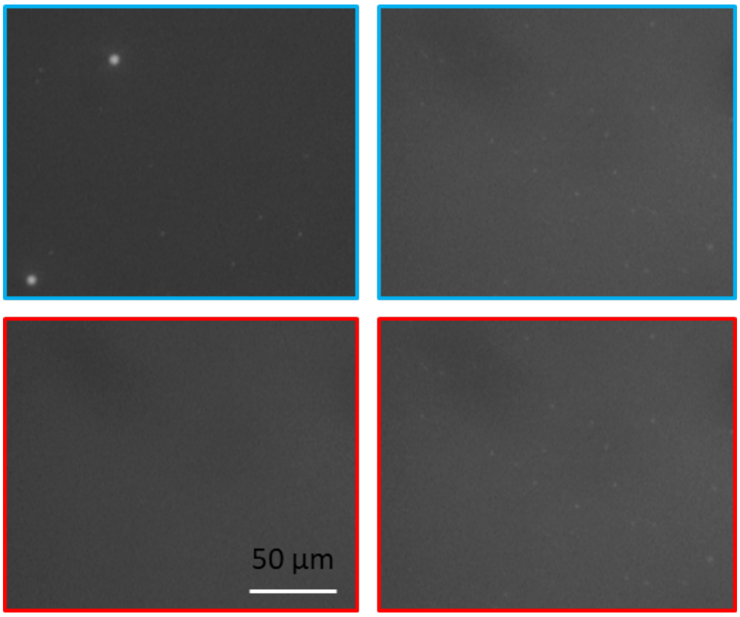

Figure S7. Epifluorescence images of POPC+TO film over PBS subphase containing CKC (3.6x10^{-6} M) at the pressure 19.3 mN/m (left column) and 35.5 mN/m (right column). Red frame represents polar (PL) and blue nonpolar (NPL) components of the film. At lower lateral pressure, small structures complementary in PL and NPL images are visible; this corresponds to aggregation of nonpolar lipids (Ref. [17]). At increased lateral pressure, small bright features complementary in polar and nonpolar images arise, indicating formation of mixed polar-nonpolar lipid aggregates.

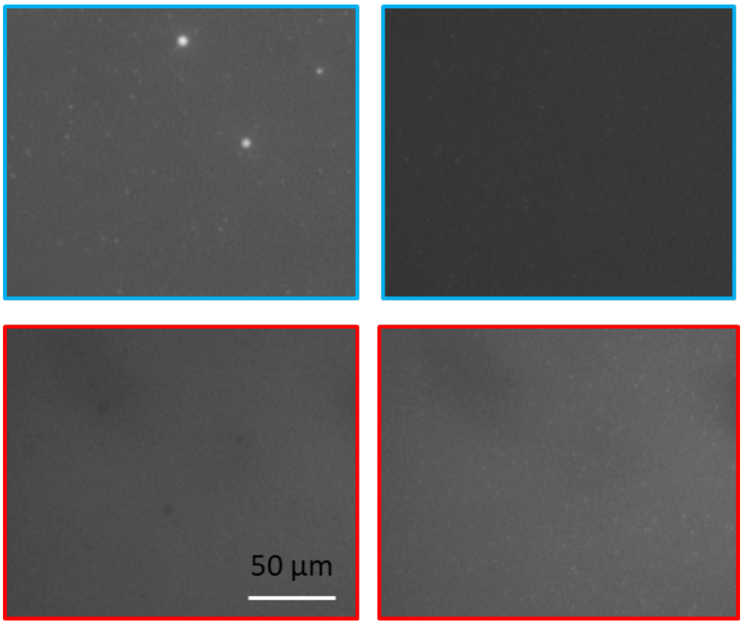

Figure S8. Epifluorescence images of POPC+TO film over PBS subphase containing P188 (10^{-6} M) recorded at pressure 28 mN/m (left column) and 35 mN/m (right column). Red frame represents polar (PL) and blue nonpolar (NPL) components of the film. At lower lateral pressure, small structures complementary in PL and NPL parts are visible; this corresponds to aggregation of nonpolar lipids. At increased lateral pressure, small bright features complementary in polar and nonpolar phases arise, indicating formation of mixed polar-nonpolar lipid aggregates.

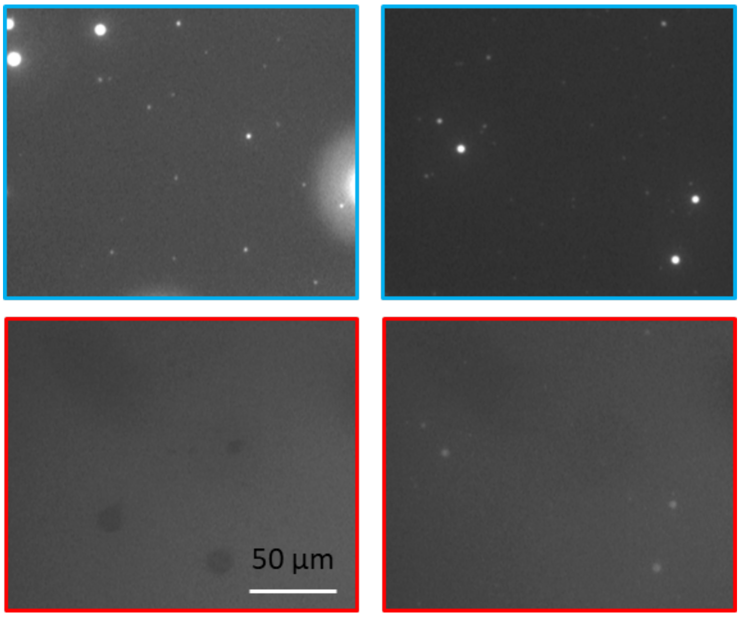

Figure S9. Epifluorescence images of POPC+TO film over PBS subphase containing P188 (10^{-6} M) and CKC ($3.6x10^{-6}$ M) at the pressure 26.5 mN/m (left column) and 36 mN/m (right column). Red frame represents polar (PL) and blue nonpolar components of the film (NPL). At lower lateral pressure, small structures complementary in PL and NPL images are visible; this indicates aggregation of nonpolar lipids. At increased lateral pressure, small bright features complementary in polar and nonpolar phases arise, indicating formation of mixed polar-nonpolar lipid aggregates. Overall, the influence of CKC and P188 is not well-pronounced in the mixed systems.

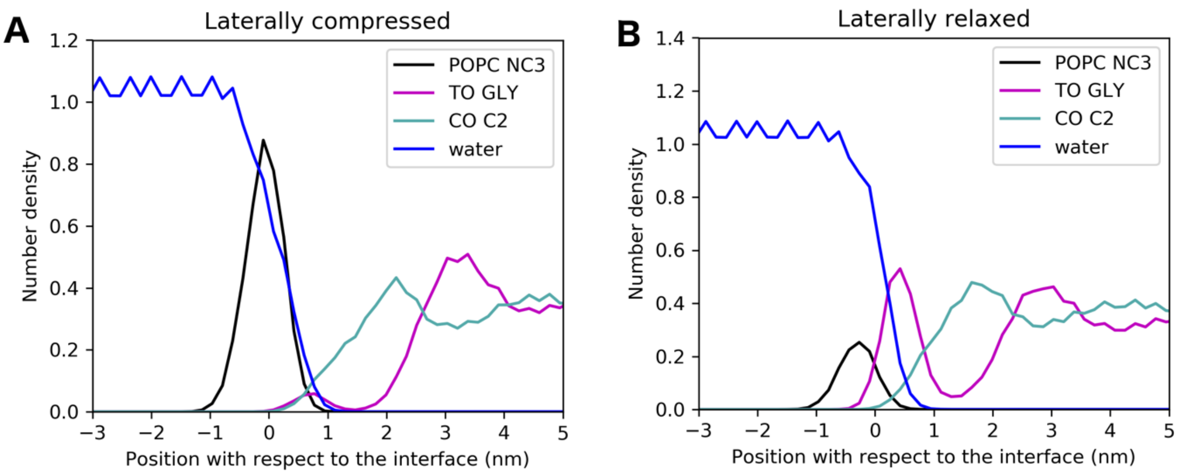

Figure S10. Density profiles of selected TFLL model components calculated in simulated films with area per polar lipid APPL=91 Å^2 (designated as compressed) (A), and 354 Å^2 (designated as relaxed) (B) in the pure lipid systems (i.e., without CKC and P188) at different APPL values. Number densities of water, POPC choline headgroup (“POPC NC3”), triglyceride glycerol backbone (“TO GLY”), and cholesterol oleate short chain terminal (“CO C2”) are depicted. Left-hand side of each plot represents aqueous tear subphase whereas the right-hand side corresponds to the nonpolar sublayer of TFLL. The data for the second lipid-air interface present in the simulation box (not shown here) are virtually the same.

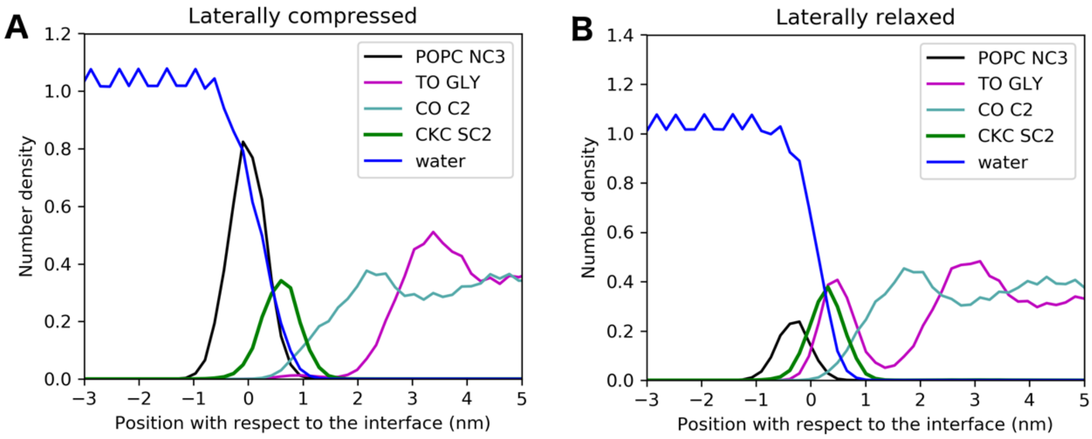

Figure S11. Density profiles of selected components of TFLL model calculated in simulated films with area per polar lipid APPL=91 Å^2 (designated as compressed) (A), and 354 Å^2 (designated as relaxed) (B) in the presence of CKC. Number densities of water, POPC polar headgroup (“POPC NC3”), triglyceride glycerol backbone (“TO GLY”), cholesterol oleate short chain terminal (“CO C2”), and CKC polar headgroup (“CKC SC2”) are depicted. Left-hand side of each plot represents aqueous tear subphase whereas the right-hand side corresponds to the nonpolar sublayer of TFLL. The data for the second lipid-air interface present in the simulation box (not shown here) are virtually the same.

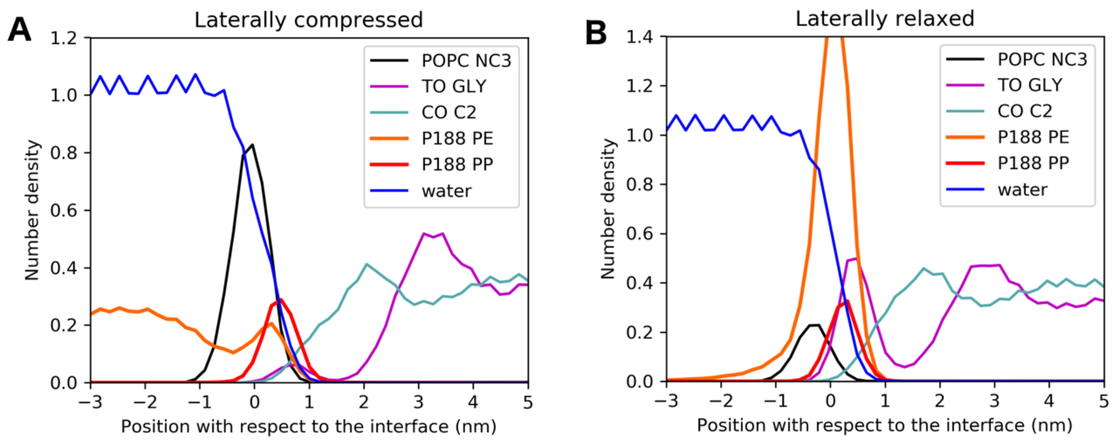

Figure S12. Density profiles of selected components of TFLL model calculated in simulated films with area per polar lipid APPL=91 Å^2 (designated as compressed) (A), and 354 Å^2 (designated as relaxed) (B)in systems at different APPL values in the presence of P188. Number densities of water, POPC polar headgroup ("POPC NC3"), triglyceride glycerol backbone ("TO GLY"), cholesterol oleate short chain terminal ("CO C2"), P188 nonpolar core ("P188 PE"), and P188 polar tails ("P188 PP") are depicted. Left-hand side of each plot represents aqueous tear subphase whereas the right-hand side corresponds to the nonpolar sublayer of TFLL. The data for the second lipid-air interface present in the simulation box (not shown here) are virtually the same.

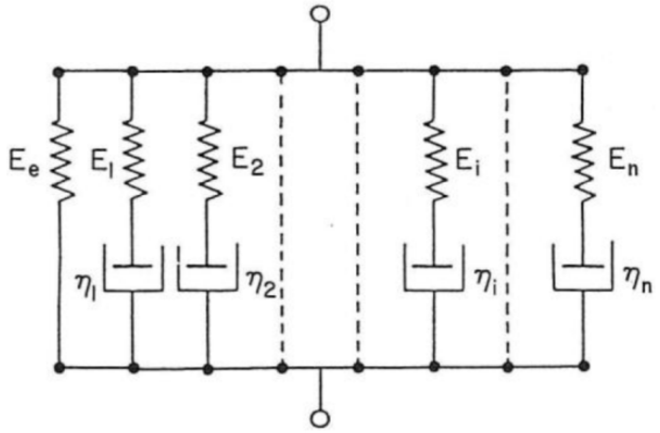

Figure S13. A schematic representation of the Maxwell models describing the output of the equation $\Delta\pi = A_i \exp \sum\left(-\frac{t}{\tau_i}\right) + \Delta\pi_{EQ}$.

MD simulation details

Simulations were performed employing a periodic box with a slab of water in the middle of the box. As typical in MD simulations of water-air films, the box was elongated in one direction so that two water-air interfaces were formed. The resulting box has the size of ~470 × 470 × 1040 $Å^2$. Each interface was covered by a lipid film of the same composition. The presence of two interfaces allows for maintaining box symmetry as well as for obtaining better statistics because two system replicas are included in a single simulation. In the performed analysis, the results obtained for each replica were virtually identical. Total numbers of lipids in the box (including both interfaces) are given in Table S1. The number of polar lipids was chosen such that a desired APPL (area per polar lipid, defined as the surface area of one interface, in each system equal to 470 × 470 $Å^2$, divided by the number of polar lipids per one interface). Molecules of CKC and P188 were added to selected systems, as described in the main manuscript. All MD simulations were performed using GROMACS 5.1 software package. The 1.1 nm cut-off was use for non-bonded interactions treated with the potential-shift-Verlet method. Long-range electrostatics was treated with the reaction-field algorithm with the relative electrostatic screening parameter of 15. Newton equations of motions were integrated with 10 fs time step. The temperature of 305 K was set and controlled vie the velocity rescale algorithm with 1.0 ps coupling constant. Bond length in MARTINI molecules were kept constant using the LINCS algorithm. Simulations were performed with the fixed box size. Trajectories of 2000 ns were calculated, and the first 1000 ns was treated as equilibration. A stabilization of numbers of contacts between different molecule types (e.g., polar lipids-water, nonpolar-polar lipids) was used as the equilibration criterion. In-house force field parameters for ceramide and CKC molecules are listed below (in GROMACS format).

Table S1. Compositions of simulated systems. Numbers of lipids, water, CKC and P188 molecules used in laterally relaxed and compressed systems. CKC and P188 were absent in simulation runs of pure lipid films, and one of them was absent in simulations including only one non-lipid component. Chloride anions were added to the water phase in the systems containing CKC to neutralize the charge. Note that each water bead in the MARTINI model represents four actual water molecules. Area per polar lipid (APPL) and CKC to polar lipid molar ratio (CKC:POLAR) are also given.

	laterally relaxed	laterally compressed
POPC	180	686
POPE	64	256
PPCS	16	64
PPCE	16	64
CKC	290	290
Cl^-	290	290
TO	2544	2544
CO	2400	2400
P188	8	8
water beads	72000	72000
APPL (Å^2)	354	91
CKC:POLAR	1.05	0.27

Force field parameters of the employed ceramide and CKC molecules (in Gromacs itp format).

Ceramide

```
[moleculetype]
; molname     nrexcl
  PPCE        1

[atoms]
; id   type  resnr  residu  atom  cgnr  charge
   1   P1    1      PPCE    OH1   1     0
   2   P5    1      PPCE    AM1   2     0
   3   P1    1      PPCE    AM2   3     0
   4   C1    1      PPCE    C1A   4     0
   5   C1    1      PPCE    C2A   5     0
   6   C1    1      PPCE    C3A   6     0
   7   C1    1      PPCE    C4A   7     0
   8   C3    1      PPCE    D1B   8     0
   9   C1    1      PPCE    C2B   9     0
  10   C1    1      PPCE    C3B   10    0
  11   C1    1      PPCE    C4B   11    0

[bonds]
; i j   funct  length  force.c.
  1 2   1      0.37    1250
  2 3   1      0.37    1250
  2 4   1      0.47    1250
  4 5   1      0.47    1250
  5 6   1      0.47    1250
  6 7   1      0.47    1250
  3 8   1      0.47    1250
  8 9   1      0.47    1250
  9 10  1      0.47    1250
  10 11 1      0.47    1250

[angles]
; i j k       funct  angle  force.c.
  1 2 3       2      120.0  25.0
  1 2 4       2      180.0  25.0
  2 4 5       2      180.0  25.0
  4 5 6       2      180.0  25.0
  5 6 7       2      180.0  25.0
  3 8 9       2      180.0  45.0
  8 9 10      2      180.0  25.0
  9 10 11     2      180.0  25.0
```

CKC

[moleculetype]
; molname nrexcl
CKC 1

[atoms]
;id type resnr residu atom cgnr charge
1 SC5 1 CKC SC1 1 0
2 SC5 1 CKC SC2 2 0
3 SC5 1 CKC SC3 3 0
4 Q0 1 CKC NC3 4 1.0
5 C1 1 CKC C1 5 0
6 C1 1 CKC C2 6 0
7 C1 1 CKC C3 7 0
8 C1 1 CKC C4 8 0

[bonds]
; i j funct length force.c.
3 4 1 0.47 1250
4 5 1 0.47 1250
5 6 1 0.47 1250
6 7 1 0.47 1250
7 8 1 0.47 1250

[constraints]
; i j funct length
3 1 1 0.27
3 2 1 0.27
1 2 1 0.27

[angles]
; i j k funct angle force.c.
4 3 1 2 150.0 50.0
4 3 2 2 150.0 50.0
3 4 5 2 180.0 25.0
4 5 6 2 180.0 25.0
5 6 7 2 180.0 25.0
6 7 8 2 180.0 25.0

[dihedrals]
; i j k l funct angle force.c.
4 1 2 3 2 0.0 50.0 ; to prevent backflipping of ring
